# Early Insights Among Emergency Medicine Physicians on Artificial Intelligence: A National, Convenience-sample Survey of the American College of Emergency Physicians

**DOI:** 10.1016/j.acepjo.2025.100308

**Published:** 2025-12-26

**Authors:** Bradley D. Shy, Cristiana Baloescu, Isaac V. Faustino, R. Andrew Taylor, Michael Gottlieb, Rohit B. Sangal, Colton Hood, Nicholas Genes, Elaine J. Rabin, The ACEP AI Task Force

**Affiliations:** 1 Department of Emergency Medicine, University of Colorado, School of Medicine, Aurora, Colorado, USA; 2 Department of Emergency Medicine, Yale School of Medicine, New Haven, Connecticut, USA; 3 Department of Emergency Medicine, University of Virginia School of Medicine, Charlottesville, Virginia, USA; 4 Department of Emergency Medicine, Rush University Medical Center, Chicago, Illinois, USA; 5 Department of Emergency Medicine, George Washington University School of Medicine and Health Science, Washington, DC, USA; 6 Ronald O. Perelman Department of Emergency Medicine, NYU Langone Health, New York, New York, USA; 7 NYU Grossman School of Medicine, Department of Emergency Medicine, New York, New York, USA; 8 Department of Emergency Medicine, Icahn School of Medicine at Mount Sinai, New York, New York, USA

**Keywords:** AI, artificial intelligence, emergency medicine, algorithmic bias, integration, physician attitudes, professional support

## Abstract

**Objectives:**

This study aimed to assess the current utilization of artificial intelligence (AI) tools among emergency physicians, their attitudes toward AI in clinical practice, and how a national physician professional organization could best support its members regarding AI.

**Methods:**

A cross-sectional survey was emailed to American College of Emergency Physicians members and made available to conference attendees at a national symposium. The survey collected demographic information, details on the use of noninstitutional and institutional AI tools, attitudes toward AI, and desired forms of support. Descriptive statistics were used to summarize the data.

**Results:**

A total of 658 physicians responded, primarily practicing attendings (78%) and residents (9%), with 60% aged 35 to 54 years and 67% identifying as male; these respondents represented 2% of the membership of American College of Emergency Physicians. Noninstitutional AI tool use (eg, ChatGPT and independent electrocardiogram interpretation) was reported by 31% of respondents. Institutional AI was integrated into 52% of respondents’ practices, with 18% regularly using ambient AI documentation and 22% using AI-assisted clinical decision support. AI tools for point-of-care ultrasound were available to 10%, and AI-assisted radiology interpretation was used by 14%, mainly for X-rays and computed tomography. Operational AI for triage, capacity management, and staff optimization were reported by 15%, while 9% used AI-assisted coding and billing. Moreover, 75% believed AI improves clinical efficiency, 57% felt it enhanced care quality, but 12% expressed concern about job displacement, 16% are unsure whether AI tools would adequately comply with Health Insurance Portability and Accountability Act regulations, and 38% noted potential biases. About half desire educational support and guidelines.

**Conclusion:**

In this nonrepresentative emergency physician survey, respondents reported moderate rates of adoption of AI tools and generally positive attitudes toward AI’s impact on efficiency and care quality. However, respondents also reported important concerns about job displacement, Health Insurance Portability and Accountability Act regulation compliance, and potential biases. Larger studies are needed to more fully understand emergency physician views on AI.

**The Bottom Line**

We surveyed emergency physicians to understand their current attitudes toward, and use of, artificial intelligence (AI). Notably, 52% reported that their institution provides AI tools; 18% regularly use it for documentation; 22% use it for clinical decision support; and 31% reported noninstitutional tool use. Most responding emergency physicians expect efficiency and quality of care to improve, but over one-third expressed concern about bias perpetuation. Fewer respondents worry about Health Insurance Portability and Accountability Act or job displacement issues. Overall, emergency physicians were found to have heterogeneous views and experiences regarding the emergence of AI.

## 1 Introduction

### 1.1 Background

Artificial intelligence (AI) has the potential to impact diagnostic accuracy, clinical decision making, and operational efficiency across many medical specialties.^1^ In particular, AI applications—ranging from advanced imaging interpretation to predictive analytics to natural language processing and autonomous clinical decision support—are expected to address challenges inherent in health care delivery.^2^ In emergency medicine (EM), the rapid growth of machine learning and AI-based algorithms are already being studied as a tool to assist in diagnostics, treatment plans, and managing complex clinical data.^3^, ^4^, ^5^ However, some have expressed concerns regarding the potential effects of AI on medical practice, labor force demands, protected health information privacy, and perpetuation of existing clinical biases.^6^^,^^7^ With the continued advancement and integration of AI technology into the daily practice of clinical EM, it is increasingly vital for national organizations to support physicians in effectively and appropriately using AI in clinical settings. The American College of Emergency Physicians (ACEP), a professional organization representing over 38,000 emergency physicians (EPs), established the ACEP AI Taskforce to examine the potential impacts of AI on the field and develop recommendations to support EM physicians as AI use grows.^8^

### 1.2 Importance

AI tools may play a crucial role in improving patient care. However, AI presents ethical and operational challenges and risks. Little is known about how extensively these tools are integrated into EM practice and what attitudes EPs hold toward their implementation.

### 1.3 Goals of This Investigation

This cross-sectional survey study aimed to describe the current use of AI tools by EPs in clinical practice, provided by their institutions and otherwise. Furthermore, we sought to capture attitudes toward AI in clinical care and identify areas in need of expanded resources, training, and advocacy.

## 2 Methods

### 2.1 Study Design

A specially convened ACEP AI Task Force, composed of EP leaders and AI content experts, initially designed the survey by reviewing the literature, existing tools, and engaging experts to establish content validity. The survey was subsequently piloted using a think-aloud technique to gather response process validity.^9^ The survey tool was refined based upon this feedback. Initial survey instrument questions were reformatted into a standardized Likert format and ambiguous language regarding AI tool usage was removed. The entire task force then completed a final consensus review prior to being sent to participants. A member of the Taskforce drafted the survey using a survey platform (Qualtrics).

### 2.2 Setting and Selection of Participants

Survey collection was conducted between September 27, 2024, and February 10, 2025. The survey was distributed via email to all ACEP members, with promotion at the 2024 ACEP Scientific Assembly in Las Vegas, Nevada, attended by approximately 6000 participants. While non-EP conference attendees were allowed to complete the survey, this study only analyzed responses from EPs. Multiple reminder emails were sent to the general membership as well as to members of 32 of the organization’s committees and sections to encourage participation (Supplementary Appendix 1).

### 2.3 Measurements

The survey instrument (Supplementary Appendix 2) included 32 questions designed to capture information on demographics, clinical use of AI tools, and attitudes toward AI adoption in EM. The survey solicited the following types of information:

1. Physician characteristics and primary site demographics: current role/training (eg, attending, resident, and fellow), gender, race/ethnicity, age, and hospital characteristics (eg, rural and academic).
2. Clinical use of AI tools: clinical decision support, information management, radiology interpretation including point-of-care ultrasound, ambient documentation, coding/billing, and emergency department (ED) operations.
3. EP attitudes toward AI (eg, AI’s predicted effect on future ED efficiency and predicted impact on ED workforce).
4. Desired support from a national organization (eg, educational resources and advocacy).

Most survey questions were mandatory for survey respondents so data missingness was minimal; for the minority of optional survey questions, we reported “N/A” for survey respondents that did not respond.

### 2.4 Data Analyses

Descriptive statistical techniques were applied to summarize the data set. Categorical variables are reported as frequencies and percentages as appropriate. ACEP provided gender and race/ethnicity membership demographics for comparison with survey respondents.

### 2.5 Ethical Approval

This research was classified as exempt by the Colorado Multiple Institutional Review Board.

## 3 Results

### 3.1 Demographics of Respondents

**Figure 1:**
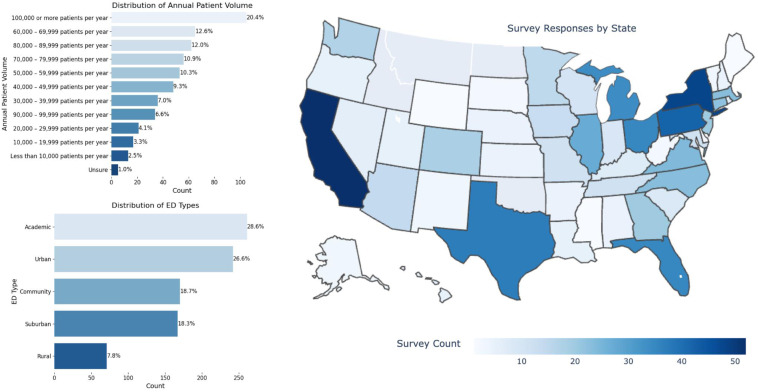
Geographical and site information for physician respondents. ED, emergency department.

A total of 658 ACEP physicians responded to the survey, representing 1.8% of the ACEP membership. The majority of respondents were practicing EM attending physicians (78%), identified as male (67%), and were white/Caucasian (78%). A majority of respondents (53%) were aged 44 years or younger. A summary of the survey respondent demographics—as well as corresponding ACEP membership demographics for gender, race/ethnicity, and training level—are shown in Table 1. Size, location, and hospital type of respondents’ hospitals are shown in Figure 1.

**Table 1:** Respondent demographics and comparison to ACEP membership demographics.

Survey category	Survey (N = 658)	Survey (%)	ACEP membership (%)
Current work/training			
Practicing EM attending physicians	511	78	48
Fellows	28	4	1
Residents	58	9	25
Other	61	9	16
Gender			
Male	441	67	55
Female	207	31	32
Other/no response	10	2	13
Race/ethnicity			
White	503	77	36
Black or African American	21	3	3
Asian	74	11	6
Hispanic or Latino/Latina	45	7	4
Native American, Alaska Native, Native Hawaiian or Other Pacific Islander	2	<1	<1
Other/no response	50	8	52
Multiracial	8	1	2
Age group (y)			
18-24	0	0	Not available
25-34	119	18	
35-44	228	35	
45-54	165	25	
55-64	78	12	
65-74	56	9	
75-84	11	2	
NA	1	0

### 3.2 Use of AI Tools

Regarding the AI tool use, 61% of respondents reported using at least some AI tools in their clinical work, with 31% using tools not integrated into their institutional systems, such as independent electrocardiogram (EKG) interpretation (41%) or natural language processing applications like ChatGPT (63%). Further, 52% of respondents worked in health systems where AI was integrated into clinical workflows, with common applications including clinical decision support (22%), radiology interpretation (14%), and ambient documentation (18%).

### 3.3 Attitudes toward AI

This study showed that 75% of respondents agreed that AI would improve clinical efficiency in EM and that 57% believed that it would enhance the quality of care for ED patients. However, 12% expressed concerns about AI potentially reducing the number of EM physicians needed, and 16% were unsure whether AI tools would adequately comply with Health Insurance Portability and Accountability Act regulations. Bias in AI systems was a concern for 38% of respondents.

### 3.4 Desired Professional Society Efforts

**Figure 2:**
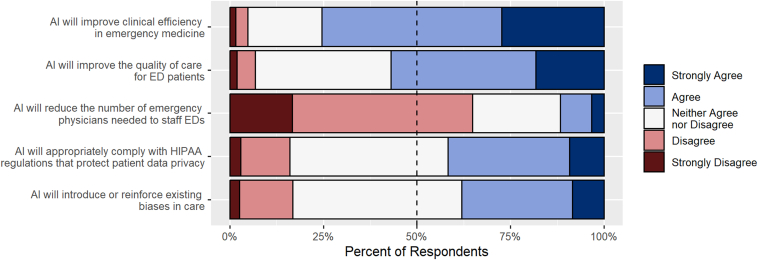
Emergency physician respondent reported attitudes toward artificial intelligence. ED, emergency department; HIPAA, Health Insurance Portability and Accountability Act.

**Figure 3:**
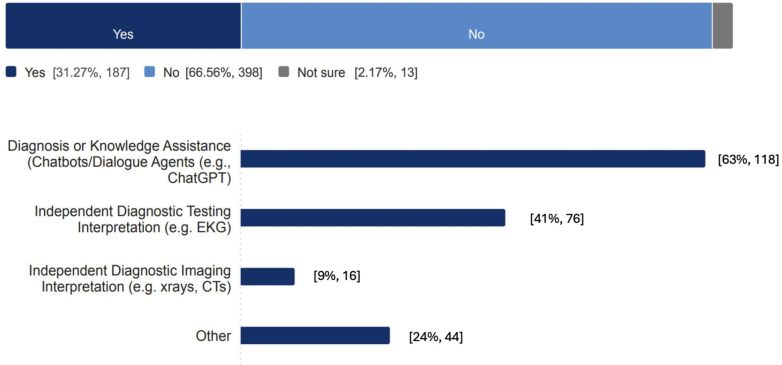
Use of noninstitutionally integrated artificial intelligence tools in clinical work. CT, computed tomography; EKG, electrocardiogram.

EP respondents requested lists of available tools (60%), policies and consensus guidance (52%), and educational resources (51%) as the most needed resources from ACEP. Summary data of respondents’ reported use of institutionally integrated AI tools attitudes toward AI and use of noninstitutionally integrated AI tools in clinical work are depicted in Table 2, Figure 2, and Figure 3 respectively. In the appendices, we describe EP use of AI by age, training level and hospital type (Supplementary Appendix 3), EP attitude toward AI by age, training level and hospital type (Supplementary Appendix 4); and preference for professional organization resource support regarding AI (Supplementary Appendix 5).

**Table 2:** Physician reported availability and use of AI tools integrated into health system (N = 658).

Survey category	Yes	No/not sure/NA
Clinical decision support		
Clinical decision support is available	127 (19.3)	No: 413 (62.8)
		Not sure: 42 (6.4)
		NA: 76 (11.5)
Information management	18 (2.7)	640 (97.3)
Diagnosis	29 (4.4)	629 (95.6)
Treatment planning	59 (9.0)	599 (91.0)
Outcome/risk assessment	66 (10.0)	592 (90.0)
Other	25 (3.8)	633 (96.2)
Radiology interpretation		
Radiology interpretation is available	84 (12.8)	No: 453 (68.8)
		Not sure: 51 (7.8)
		NA: 70 (10.6)
CT	70 (10.6)	588 (89.4)
X-ray	29 (4.4)	629 (95.6)
MRI	9 (1.4)	649 (98.6)
Ultrasound (non-POCUS)	3 (0.5)	655 (99.5)
Other	3 (0.5)	655 (99.5)
Ambient documentation		
Ambient documentation is available	108 (16.4)	No: 471 (71.6)
		Not sure: 14 (2.1)
		NA: 65 (9.9)
AI for POCUS		
AI for POCUS is available	56 (8.5)	No: 498 (75.7)
		Not sure: 35 (5.3)
		NA: 69 (10.5)
Interpretation of findings	45 (6.8)	613 (93.2)
Autolabeling structures	20 (3.0)	638 (97.0)
Guiding hand movements to improve image optimization	15 (2.3)	643 (97.7)
Coding/billing		
AI-assisted coding/billing used by the department	50 (7.6)	No: 290 (44.1)
		Not sure: 240 (36.5)
		NA: 78 (11.8)
ED operations		
AI-assisted tools used for ED operations in the health care system	91 (13.8)	No: 141 (21.4)
		Not sure: 380 (57.8)
		NA: 46 (7.0)
Triage	26 (4.0)	632 (96.0)
Boarding/capacity management	30 (4.6)	628 (95.4)
Staffing optimization (scheduling)	21 (3.2)	637 (96.8)
Other	32 (4.9)	626 (95.1)

AI, artificial intelligence; CT, computed tomography; ED, emergency department; MRI, magnetic resonance imaging; POCUS, point-of-care ultrasound.

## 4 Limitations

This study is subject to several limitations. Notably, only 2% of ACEP members responded, and respondents were not demographically representative of organizational membership. The survey methodology was susceptible to nonresponse bias and self-selection bias as physicians more interested in AI, or more concerned about its effects, may have been more likely to respond. A high level of missingness in the demographic data of the ACEP general membership precluded a meaningful comparison assessing how representative the survey respondents were of the organization’s membership. Different demographic groups of EPs might have attitudes toward AI adoption that were not reflected in this survey. The survey also relied on self-reported data by individuals who may not be fully informed of institutional resources. This may introduce bias in estimating the prevalence of AI tool usage. The cross-sectional nature of the survey also limits our ability to assess temporal trends in AI adoption, which are likely very dynamic.

## 5 Discussion

This national survey provides insights into the current landscape of AI adoption in EM and highlights both enthusiasm and concerns among EM physicians. The relatively high rates of independent AI tool use outside health systems suggest that many physicians are exploring AI’s potential, but institutional integration remains inconsistent. While most respondents were optimistic about AI’s role in improving clinical efficiency and patient care, widespread concerns regarding job displacement, data privacy, and algorithmic bias reflect broader ethical considerations. These mixed attitudes toward AI of EPs mirror similar perspectives of physicians in other specialities.^10^, ^11^, ^12^, ^13^, ^14^

In the near future, a variety of new applications of EM AI have been suggested throughout the patient’s care journey.^12^ As AI expands in EM and its positive and negative effects proliferate, there is an urgent need for structured training and clear guidelines on its use. Specifically, in this current survey, EPs identified lists of AI tools as a desired priority for ACEP. Other specialities such as neuroradiology have developed checklists for evaluating AI tools and more general competency checklists have been designed for physicians using such AI tools.^15^^,^^16^ ACEP and similar EM organizations are well-positioned to lead these efforts by developing AI-specific educational modules, advocating for AI equity, and ensuring that AI tools are designed to align with Health Insurance Portability and Accountability Act and other regulatory standards.

Further longitudinal research should evaluate AI integration in EM over time and explore the evolution of AI’s effects on clinical outcomes, efficiency, labor markets, data privacy, bias, and perpetuation of existing biases. Moreover, there is a need for the development of educational programs and research assessing their effectiveness in improving AI literacy among EPs. As AI adoption is likely to increase rapidly and the current study is a point-in-time description, repeated analysis will be needed to evaluate the evolving use of AI in EM.^16^ Developing scalable, ethical, and bias-resistant AI tools for emergency settings should remain a priority.^17^

In conclusion, our survey describes several hypothesis-generating findings, which may be further elucidated with larger, more representative surveys or other research modalities. This current survey highlights both the growing interest in AI among EM physicians and the need for more structured support in integrating these tools into clinical practice. While AI is viewed as having the potential to enhance efficiency and patient care, concerns about data privacy, bias, and job displacement must be addressed. National organizations such as ACEP may play a pivotal role in providing resources, education, and advocacy to ensure that AI is ethically and effectively incorporated into EM.

## Author Contributions

All authors helped to conceive and design the study. IVF and CB abstracted and analyzed the data. BDS, CB, and RAT drafted the manuscript. All authors reviewed and approved the final version of the manuscript. RBS and EJR take responsibility for the manuscript as a whole.

## Funding and Support

Administrative support for this survey was provided by the American College of Emergency Physicians.

## Conflict of Interest

RAT receives grant support from Beckman Coulter Inc, for AI development and evaluation. CB receives grant support from Philips and GE for development and evaluation of AI for ultrasound. The other authors declare that they have no known competing financial interests or personal relationships that could have appeared to influence the work reported in this paper.

## Supplemental material

Supplementaty Material
