# Supplementary material for: Early Insights Among Emergency Medicine Physicians on Artificial Intelligence: A National, Convenience-sample Survey of the American College of Emergency Physicians
Source: J Am Coll Emerg Physicians Open. 2025 Dec 26;7(1):100308. doi: 10.1016/j.acepjo.2025.100308 (PMC12796722; doi:10.1016/j.acepjo.2025.100308)
Supplement: Supplementaty Material [file mmc1.docx]

**Supplementary Appendix 1: List of American College of Emergency Physicians (ACEP) committees and sections solicited for survey completion**

Academic Affairs Committee
Air Medical Transport Section
American Association of Women Emergency Physicians Section
Careers in Emergency Medicine Section
Coding and Nomenclature Advisory Committee
Critical Care Medicine Section
Disaster Medicine Section
Diversity, Equity and Inclusion Committee
Education Committee
EM Practice Committee
Emergency Medical Services-Prehospital Care Section
Emergency Medicine Practice Management and Health Policy Section
Emergency Medicine Workforce Section
Emergency Telehealth Section
Ethics Committee
Health Information Technology Committee
International Emergency Medicine Section
Medical Directors Committee
Medical Legal Committee
Pediatric Emergency Medicine Committee
Pediatric Emergency Medicine Section
Quality and Patient Safety Committee
Quality Improvement & Patient Safety Section
Reimbursement Committee
Research Committee
Rural Emergency Medicine Section
State Legislative/Regulatory Committee
Tactical and Law Enforcement Medicine Section
Ultrasound Committee
Wellness Section
Wilderness Medicine Section
Young Physicians Committee

**Supplementary Appendix 2: Survey instrument**

**Introduction**

Your responses will help ACEP better support its members with AI-related resources, education, and advocacy efforts. The survey will take approximately 5 minutes.

Artificial intelligence is broadly defined as the capability of a machine to imitate intelligent human behavior. The term encompasses systems or machines that display forms of:

- autonomy

- adaptivity

- ability to process information and make decisions based on this information.

AI systems can perform tasks that would typically require human intelligence, such as understanding natural language, recognizing patterns, solving problems, and learning from past experiences.

**Physician Characteristics and Primary Site Demographics**

Which best describes your current work/training?

- Medical student
- Resident
- Fellow
- Practicing EM Attending
- Non-clinical EM Physician
- Retired Physician
- Non-Physician Practitioner (Physician Assistant or Nursing Practitioner)
- Other

Gender

- Male
- Female
- Prefer to self-describe
- Prefer not to say

Please indicate the US Census 'race' category(ies) with which you identify. Please select all that apply.

- White or Caucasian
- Black or African American
- Asian
- American Indian or Alaska Native
- Native Hawaiian or Pacific Island
- Prefer not to answer

Are you of Hispanic, Latino/Latina, or Latinx origin?

- Yes
- No
- Prefer not answer

What is your primary ED's estimated annual volume?

[free text]

What type of hospital do you work in? Please select all that apply.

- Rural
- Suburban
- Urban
- Academic
- Community

In what state/region/area do you primarily work/live?

[list of countries/states]

**Non-institutional clinical uses**

In your clinical work, are you personally using AI tools NOT integrated into your institution (e.g. ChatGPT, independent EKG interpretation, etc)?

- Yes
- No
- Not sure

What AI tools are you independently using that are NOT integrated into your institution for clinical work? Please

select all that apply:

- Diagnosis or Knowledge Assistance (Chatbots/Dialogue Agents (e.g.,ChatGPT)
- Independent Diagnostic Testing Interpretation (e.g. EKG)
- Independent Diagnostic Imaging Interpretation (e.g. xrays, CTs)
- Other

Is ambient AI documentation available in your emergency department?

*Definition: Ambient AI Documentation is technology that passively records clinical conversations with patients or*

*other medical staff and creates an organized clinical note of the ED visit from these conversations, not just a*

*dictation.*

- Yes
- No
- Unsure

How often do you use Ambient AI for documentation?

- Never
- Rarely
- Sometimes
- Often
- Always

Is AI-assisted Clinical Decision Support available in your emergency department? (Please select all that

apply):

- Information management (e.g., prior note summarization)
- Diagnosis (e.g. automated differential diagnosis generation, risk of particular diagnosis)
- Treatment Planning (e.g, drug interaction risk, antibiotic resistance prediction)
- Outcome risk assessment/prognosis (e.g., risk of deterioration, risk of return visits, death)
- Other
- None
- Unsure

How often per shift do you use AI-assisted clinical decision aides?

- Never
- Rarely
- Sometimes
- Often
- Always

Is AI-assistance available for Point-of-care Ultrasound (POCUS) in your emergency department?

- Yes
- No
- Unsure

Are any of the following types of AI assistance available for use with Point-of-care Ultrasound (POCUS) in your

emergency department? (Please select all that apply):

- Guiding Hand movements to improve image optimization
- Auto-labeling structures
- Interpretation of findings (example: calculation of ejection fraction)
- POCUS is not used in my ED
- Other

How often do you use AI-assistance for POCUS?

- Never
- Rarely
- Sometimes
- Often
- Always

Is AI-assisted radiology interpretation (e.g., CT, MRI, xray) available in your emergency department to facilitate

decision-making by emergency physicians?

- Yes
- No
- Not Sure

Please select all available AI-assisted radiology modalities you are aware of (Please select all that

apply):

- CT
- MRI
- Xray
- Ultrasound (non-POCUS)
- Other

If AI-generated radiology interpretations are available, how often per shift do you encounter them?

- Never
- Rarely
- Sometimes
- Often
- Always
- Not available

Are the following AI diagnostic tools available in your emergency department? (Please select all that

apply):

- EKG interpretation (beyond the basic interpretation performed by the machine)
- EEG interpretation
- Lab interpretation
- Other
- None

Are AI-assisted tool(s) used for ED operations in your healthcare system? (Please select all that apply):

- Triage
- Boarding/Capacity management
- Staffing Optimization (e.g., nursing/physician scheduling)
- Other
- None

Does your department use AI-assisted coding/billing? *Example: recommending an E + M code or automatic*

*queries for documentation adjustment.*

- Yes
- No
- Not sure

What other AI tools does your hospital's health system use that we did not list?

[free text]

Please rate the degree to which you agree or disagree with each of the following statements regarding the future of emergency medicine.


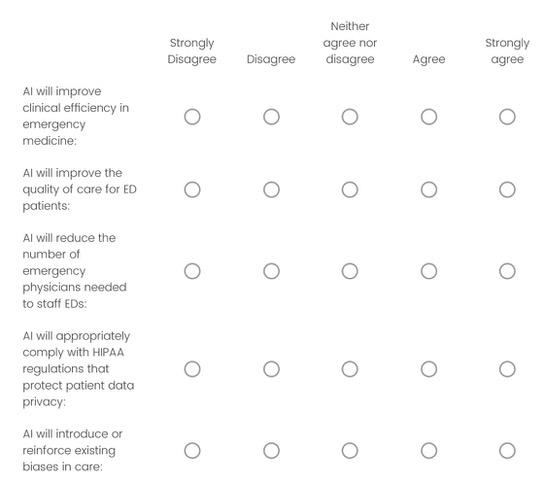


**ACEP support**

What could ACEP provide that would be most helpful in your practice of EM with AI? Please select up to 3.

- Educational resources
- Lists of available tools
- Advocacy for institutions not providing AI tools
- Healthcare equity monitoring initiatives
- Data/IT environments to develop AI tools
- Policies and Consensus Guidance

Do you have anything else you would like to say about AI, any thoughts on AI, or how ACEP can support you?

[free text]

Please enter your name and email address below (separated by a comma) if you would like to be entered into a drawing for 1 of 10, $100 GrubHub gift cards.

[free text]

**Supplementary Appendix 3: Emergency physician use of Artificial Intelligence (AI) by age, training level, and type of hospital (N = 658)**

| **AI-assisted CDS** | | Use AI tool (n, %) |
| --- | --- | --- |
| Age range | < 45 years old | 71 (10.8%) |
|  | ≥ 45 years old | 56 (8.5%) |
| Current work/training | Attending | 104 (15.8%) |
|  | Fellow | 5 (0.8%) |
|  | Resident | 14 (2.1%) |
|  | Other | 4 (0.6%) |
| Setting | Urban/Suburban | 95 (14.4%) |
|  | Rural | 6 (0.9%) |
| Hospital type | Academic | 81 (12.3%) |
|  | Community | 13 (2.0%) |
| **Radiology Interpretation** | | Uses AI tool (n, %) |
| Age range | < 45 years old | 43 (6.5%) |
|  | ≥ 45 years old | 41 (6.2%) |
| Current work/training | Attending | 70 (10.6%) |
|  | Fellow | 3 (0.5%) |
|  | Resident | 11 (1.7%) |
|  | Other | 0 (0%) |
| Setting | Urban/Suburban | 62 (9.4%) |
|  | Rural | 4 (0.6%) |
| Hospital type | Academic | 42 (6.4%) |
|  | Community | 17 (2.6%) |
| **Ambient documentation** | | Uses AI tool (n, %) |
| Age range | < 45 years old | 68 (10.3%) |
|  | ≥ 45 years old | 40 (6.1%) |
| Current work/training | Attending | 91 (13.8%) |
|  | Fellow | 6 (0.9%) |
|  | Resident | 7 (1.1%) |
|  | Other | 4 (0.6%) |
| Setting | Urban/Suburban | 86 (13.1%) |
|  | Rural | 3 (0.5%) |
| Hospital type | Academic | 61 (9.3%) |
|  | Community | 13 (2.0%) |
| **POCUS** | | Uses AI tool (n, %) |
| Age range | < 45 years old | 37 (5.6%) |
|  | ≥ 45 years old | 19 (2.9%) |
| Current work/training | Attending | 49 (7.5%) |
|  | Fellow | 0 (0%) |
|  | Resident | 7 (1.1%) |
|  | Other | 0 (0%) |
| Setting | Urban/Suburban | 44 (6.7%) |
|  | Rural | 4 (0.6%) |
| Hospital type | Academic | 37 (5.6%) |
|  | Community | 5 (0.8%) |
| **AI-diagnostic tools** | | Uses AI tool (n, %) |
| Age range | < 45 years old | 29 (4.4%) |
|  | ≥ 45 years old | 27 (4.1%) |
| Current work/training | Attending | 46 (7.0%) |
|  | Fellow | 2 (0.3%) |
|  | Resident | 5 (0.8%) |
|  | Other | 3 (0.5%) |
| Setting | Urban/Suburban | 39 (5.9%) |
|  | Rural | 0 (0%) |
| Hospital type | Academic | 32 (4.9%) |
|  | Community | 5 (0.8%) |
| **AI-assisted coding/billing** | | Uses AI tool (n, n%) |
| Age range | < 45 years old | 21 (3.2%) |
|  | ≥ 45 years old | 29 (4.4%) |
| Current work/training | Attending | 48 (7.3%) |
|  | Fellow | 0 (0%) |
|  | Resident | 1 (0.2%) |
|  | Other | 1 (0.2%) |
| Setting | Urban/Suburban | 39 (5.9%) |
|  | Rural | 2 (0.3%) |
| Hospital type | Academic | 27 (4.1%) |
|  | Community | 6 (0.9%) |
| **AI-assisted ED operations** | | Uses AI tool (n, %) |
| Age range | < 45 years old | 42 (6.4%) |
|  | ≥ 45 years old | 48 (7.3%) |
| Current work/training | Attending | 77 (11.7%) |
|  | Fellow | 4 (0.6%) |
|  | Resident | 6 (0.9%) |
|  | Other | 3 (0.5%) |
| Setting | Urban/Suburban | 57 (8.7%) |
|  | Rural | 8 (1.2%) |
| Hospital type | Academic | 49 (7.5%) |
|  | Community | 9 (1.4%) |

**Supplementary Appendix 4: Emergency physician attitudes towards Artificial Intelligence (AI) by age, training level, and type of hospital**

| **AI will improve clinical efficiency in emergency medicine** | | | | | | |
| --- | --- | --- | --- | --- | --- | --- |
|  |  | Strongly Disagree | Disagree | Neutral | Agree | Strongly Agree |
| Age range  (N = 618, NA = 40) | < 45 years old | 5 (0.8%) | 9 (1.5%) | 54 (8.7%) | 160 (25.9%) | 98 (15.9%) |
|  | ≥ 45 years old | 5 (0.8%) | 11 (1.8%) | 68 (11.0%) | 137 (22.2%) | 71 (11.5%) |
| Current work/training  (N = 618, NA = 40) | Attending | 7 (1.1%) | 17 (2.8%) | 96 (15.5%) | 246 (39.8%) | 122 (19.7%) |
|  | Fellow | 1 (0.2%) | 0 (0.0%) | 2 (0.3%) | 13 (2.1%) | 8 (1.3%) |
|  | Resident | 1 (0.2%) | 1 (0.2%) | 11 (1.8%) | 20 (3.2%) | 19 (3.1%) |
|  | Other | 1 (0.2%) | 2 (0.3%) | 13 (2.1%) | 18 (2.9%) | 20 (3.2%) |
| Setting  (N = 462, neither = 156, NA = 40) | Urban/Suburban | 4 (0.9%) | 13 (2.8%) | 89 (19.3%) | 204 (44.2%) | 109 (23.6%) |
|  | Rural | 2 (0.4%) | 0 (0.0%) | 6 (1.3%) | 24 (5.2%) | 11 (2.4%) |
| Hospital type  (N = 402, neither = 216, NA = 40) | Academic | 2 (0.5%) | 8 (2.0%) | 49 (12.2%) | 159 (39.6%) | 83 (20.6%) |
|  | Community | 1 (0.2%) | 5 (1.2%) | 19 (4.7%) | 48 (11.9%) | 28 (7.0%) |
| **AI will improve quality of care for ED patients** | | | | | | |
|  |  | Strongly Disagree | Disagree | Neutral | Agree | Strongly Agree |
| Age range  (N = 618, NA = 40) | < 45 years old | 5 (0.8%) | 13 (2.1%) | 123 (19.9%) | 121 (19.6%) | 64 (10.4%) |
|  | ≥ 45 years old | 7 (1.1%) | 17 (2.8%) | 101 (16.3%) | 118 (19.1%) | 49 (7.9%) |
| Current work/training  (N = 618, NA = 40) | Attending | 9 (1.5%) | 26 (4.2%) | 179 (29.0%) | 195 (31.6%) | 79 (12.8%) |
|  | Fellow | 1 (0.2%) | 0 (0.0%) | 6 (1.0%) | 13 (2.1%) | 4 (0.6%) |
|  | Resident | 0 (0.0%) | 2 (0.3%) | 18 (2.9%) | 16 (2.6%) | 16 (2.6%) |
|  | Other | 2 (0.3%) | 2 (0.3%) | 21 (3.4%) | 15 (2.4%) | 14 (2.3%) |
| Setting  (N = 462, neither = 156, NA = 40) | Urban/Suburban | 6 (1.3%) | 22 (4.8%) | 155 (33.5%) | 165 (35.7%) | 71 (15.4%) |
|  | Rural | 0 (0.0%) | 2 (0.4%) | 15 (3.2%) | 17 (3.7%) | 9 (1.9%) |
| Hospital type  (N = 402, neither = 216, NA = 40) | Academic | 4 (1.0%) | 12 (3.0%) | 101 (25.1%) | 132 (32.8%) | 52 (12.9%) |
|  | Community | 2 (0.5%) | 5 (1.2%) | 38 (9.5%) | 40 (10.0%) | 16 (4.0%) |
| **AI will reduce the number of emergency physicians needed to staff EDs** | | | | | | |
|  |  | Strongly Disagree | Disagree | Neutral | Agree | Strongly Agree |
| Age range  (N = 618, NA = 40) | < 45 years old | 62 (10.0%) | 151 (24.4%) | 80 (12.9%) | 19 (3.1%) | 14 (2.3%) |
|  | ≥ 45 years old | 41 (6.6%) | 147 (23.8%) | 65 (10.5%) | 32 (5.2%) | 7 (1.1%) |
| Current work/training  (N = 618, NA = 40) | Attending | 81 (13.1%) | 245 (39.6%) | 109 (17.6%) | 39 (6.3%) | 14 (2.3%) |
|  | Fellow | 2 (0.3%) | 11 (1.8%) | 5 (0.8%) | 4 (0.6%) | 2 (0.3%) |
|  | Resident | 12 (1.9%) | 19 (3.1%) | 16 (2.6%) | 3 (0.5%) | 2 (0.3%) |
|  | Other | 8 (1.3%) | 23 (3.7%) | 15 (2.4%) | 5 (0.8%) | 3 (0.5%) |
| Setting  (N = 462, neither = 156, NA = 40) | Urban/Suburban | 73 (15.8%) | 200 (43.3%) | 94 (20.3%) | 38 (8.2%) | 14 (3.0%) |
|  | Rural | 5 (1.1%) | 24 (5.2%) | 11 (2.4%) | 2 (0.4%) | 1 (0.2%) |
| Hospital type  (N = 402, neither = 216, NA = 40) | Academic | 59 (14.7%) | 142 (35.3%) | 68 (16.9%) | 23 (5.7%) | 9 (2.2%) |
|  | Community | 19 (4.7%) | 49 (12.2%) | 25 (6.2%) | 7 (1.7%) | 1 (0.2%) |
| **AI will appropriately comply with HIPAA regulations that protect patient data privacy** | | | | | | |
|  |  | Strongly Disagree | Disagree | Neutral | Agree | Strongly Agree |
| Age range  (N = 618, NA = 40) | < 45 years old | 10 (1.6%) | 47 (7.6%) | 138 (22.3%) | 102 (16.5%) | 29 (4.7%) |
|  | ≥ 45 years old | 8 (1.3%) | 34 (5.5%) | 123 (19.9%) | 99 (16.0%) | 28 (4.5%) |
| Current work/training  (N = 618, NA = 40) | Attending | 14 (2.3%) | 67 (10.8%) | 198 (32.0%) | 163 (26.4%) | 46 (7.4%) |
|  | Fellow | 1 (0.2%) | 3 (0.5%) | 10 (1.6%) | 9 (1.5%) | 1 (0.2%) |
|  | Resident | 1 (0.2%) | 6 (1.0%) | 24 (3.9%) | 16 (2.6%) | 5 (0.8%) |
|  | Other | 2 (0.3%) | 5 (0.8%) | 29 (4.7%) | 13 (2.1%) | 5 (0.8%) |
| Setting  (N = 462, neither = 156, NA = 40) | Urban/Suburban | 11 (2.4%) | 60 (13.0%) | 167 (36.1%) | 145 (31.4%) | 36 (7.8%) |
|  | Rural | 2 (0.4%) | 2 (0.4%) | 21 (4.5%) | 12 (2.6%) | 6 (1.3%) |
| Hospital type  (N = 402, neither = 216, NA = 40) | Academic | 10 (2.5%) | 42 (10.4%) | 122 (30.3%) | 98 (24.4%) | 29 (7.2%) |
|  | Community | 2 (0.5%) | 15 (3.7%) | 49 (12.2%) | 29 (7.2%) | 6 (1.5%) |
| **AI will introduce or reinforce existing biases in care** | | | | | | |
|  |  | Strongly Disagree | Disagree | Neutral | Agree | Strongly Agree |
| Age range  (N = 618, NA = 40) | < 45 years old | 7 (1.1%) | 37 (6.0%) | 149 (24.1%) | 110 (17.8%) | 23 (3.7%) |
|  | ≥ 45 years old | 9 (1.5%) | 51 (8.3%) | 130 (21.0%) | 73 (11.8%) | 29 (4.7%) |
| Current work/training  (N = 618, NA = 40) | Attending | 13 (2.1%) | 71 (11.5%) | 226 (36.6%) | 140 (22.7%) | 38 (6.1%) |
|  | Fellow | 0 (0.0%) | 1 (0.2%) | 11 (1.8%) | 10 (1.6%) | 2 (0.3%) |
|  | Resident | 1 (0.2%) | 8 (1.3%) | 22 (3.6%) | 16 (2.6%) | 5 (0.8%) |
|  | Other | 2 (0.3%) | 8 (1.3%) | 20 (3.2%) | 17 (2.8%) | 7 (1.1%) |
| Setting  (N = 462, neither = 156, NA = 40) | Urban/Suburban | 11 (2.4%) | 54 (11.7%) | 189 (40.9%) | 134 (29.0%) | 31 (6.7%) |
|  | Rural | 2 (0.4%) | 3 (0.6%) | 24 (5.2%) | 7 (1.5%) | 7 (1.5%) |
| Hospital type  (N = 402, neither = 216, NA = 40) | Academic | 6 (1.5%) | 42 (10.4%) | 133 (33.1%) | 93 (23.1%) | 27 (6.7%) |
|  | Community | 1 (0.2%) | 12 (3.0%) | 50 (12.4%) | 31 (7.7%) | 7 (1.7%) |

**Supplementary Appendix 5: Preference for professional organization resource support for emergency physicians regarding Artificial Intelligence (AI) (N = 658)**

| **Resource** | **n, %** |
| --- | --- |
| Educational resources | 337 (51%) |
| Lists of available tools | 397 (60%) |
| Advocacy for institutions not providing AI tools | 148 (23%) |
| Healthcare equity monitoring initiatives | 152 (23%) |
| Data/IT environments to develop AI tools | 206 (31%) |
| Policies and consensus guidance | 340 (52%) |
